# Effect of *Lactobacillus acidophilus *supernatants on body weight and leptin expression in rats

**DOI:** 10.1186/1472-6882-8-5

**Published:** 2008-02-19

**Authors:** Renato Sousa, Jaroslava Halper, Jian Zhang, Stephen J Lewis, Wan-I O Li

**Affiliations:** 1 Department of Pathology, College of Veterinary Medicine, The University of Georgia, Athens, GA 30602, USA; 2 Department of Physiology/Pharmacology, College of Veterinary Medicine, The University of Georgia, Athens, GA 30602, USA

## Abstract

**Background:**

*Lactobacillus *extracts and supernatants have been used as probiotics in human and veterinary medicine for their ability to enhance wound healing and immunity. Previous data from our laboratory demonstrated that *Lactobacillus *supernatant (LS) stimulated wound healing, angiogenesis and proliferation of embryonic cells after topical application. This current study shows that LS after its administration into the cerebral ventricles of male rats exerts systemic effects.

**Methods:**

The right lateral cerebral ventricle of young male rats was accessed through intracerebroventricular cannulation (ICV) under anesthesia and aseptic conditions. One group of control rats received saline solution, a second control group received 0.8 M lactic acid solution (to control for acidity of LS), and a third group received LS. The animals were sacrificed 12, 24, 48, 96 and 120 hours after the injection. Selected tissues were collected, fixed in 10% buffered formalin and used for immunohistochemistry and *in situ *hybridization. Other tissues were frozen and extracted for immunoblotting

**Results:**

LS-injected animals had a slight decrease in body weight when compared to their initial weight and to both control groups. Using immunohistochemistry and *in situ *hybridization leptin expression was studied in multiple brain sections and peripheral adipose tissue of control and LS-injected rats. Strong cytoplasmic stain was observed by both techniques in neurons of the cerebral cortex, thalamus, hypothalamus, hippocampus and, to lesser degree, in the cells of the choroid plexus in the LS-injected rats. Control animals demonstrated much less intense staining in neurons located in the same regions using immunohistochemistry and almost no staining with *in situ *hybridization technique. Adipose tissue exhibited slight presence of leptin in LS-treated animals. In contrast no immunohistochemical staining for GM-CSF and TNFα was observed in brains from control and treated rats. Western blotting showed mild increase in leptin and leptin receptors in intestines and retroperitoneal adipose tissues of LS-injected rats.

**Conclusion:**

This study demonstrates that direct administration of LS into rat CNS leads to a decrease in body weight of rats and an increase in the expression of leptin in specific areas of the brain and retroperitoneal adipose tissue.

## Background

*Lactobacilli *are non-pathogenic Gram-positive lactic acid bacteria found in the normal intestinal microflora of animals and humans [1] and are classified as probiotic agents. *Lactobacillus *derived products, including culture supernatants have been used for their wound healing and antiviral properties as they are believed to boost energy and to be effective remedies for allergies, common cold, lactose intolerance, and have also been shown to reduce cholesterol levels and the risk of colon cancer [2-4]. Supernatants of *Lactobacillus acidophilus *were also proved to be effective against *Helicobacter pylori in vitro *and *in vivo *in people and were shown to possess antimicrobial activities against *Bacillus anthracis *and *E. coli *[3]. Our previous data have shown that *Lactobacillus *supernatants (LS) promote inflammatory response during tissue repair in rodents [5], stimulate proliferation of embryonic cells [6], and that subcutaneous injections of *Lactobacillus *supernatants into the ears of rats lead to angiogenesis [5]. Using a cytokine antibody array, leptin and several other cytokines (e.g., IL-6, IL-8 and TGFβ) were detected in medium conditioned by bovine endothelial cells exposed to LS (data not shown).

This study represents a continuation of our previous work [5]. The purpose was to identify whether LS stimulates angiogenesis in rodent CNS and/or expression of leptin in rodents. Leptin, the product of the obese gene (*ob *or *lepob*) is a 16 kDa non-glycosylated protein well known for its effects on the reduction of body weight and involvement in certain aspects of wound healing, such as angiogenesis. Leptin acts in the central nervous system through binding to leptin receptors located in the hypothalamus, particularly in the arcuate nucleus and by coordinating metabolism, feeding behavior, energy balance and neuroendocrine responses [7-9]. In addition to CNS leptin is also expressed in adipocytes, placenta [10], mammary gland [11], pituitary glands [12], and stomach [13].

In experiments designed to determine whether the supernatants elicit angiogenesis in the cerebral circulation, we injected LS into the lateral cerebral ventricles of adult, normotensive Sprague-Dawley rats. A vital serendipitous finding was that the injection led to reductions in body weight without changes in body temperature or intake of food and water. This was accompanied by increased leptin expression in CNS, and intestinal and retroperitoneal adipose tissue.

## Methods

### Preparation of supernatants from Lactobacillus cultures

Briefly, cultures of *L. acidophilus *(ATCC strains 4356 and 43121) were grown in MRS broth (pH 5.5; Difco Laboratories, Detroit, MI) at 37°C for 24 hr under microaerophilic conditions. This medium contains a rich nutrient base as well as polysorbate, acetate, magnesium, and manganese, which are known to promote the growth and proliferation of *Lactobacilli*. Overnight bacterial cultures contained 2.5 × 10^8 ^colony-forming units, and these cultures were centrifuged at 10,000 g for 15 min at 4°C. The resulting supernatants were filtered through a 0.2-μm membrane filter to remove the remaining bacteria and debris, lyophilized, and then stored at -20°C. At the time of the experiments, the lyophilized LS was reconstituted with deionized water, filtered with puradiscs (0.22- μm pore size; Whatman Inc., Ann Arbor, MI) and termed LS. The pH of LS was 2.35 + 0.15, and osmolality was 320 mOsm.

### Animals

The use of animals and the animal experiments was approved by the Animal Use Committee at The University of Georgia. Thirty eight young male Sprague Dawley rats (Harlan, Sprague Dawley, Inc., Indianapolis, IN) ranging in weight from 250 to 350 g (or 6–8 weeks of age) at the beginning of the experiment were used. The animals were housed individually in a temperature-controlled room (22 ± 1°C) with a 12 h-12 h light-dark cycle. Food and water were available *ad libitum *in their cages. Body parameters such as rectal temperature and body weight were checked daily, as was food and water consumption. At the end of each experiment rats were euthanized by CO_2 _overdose.

### Intra-cerebroventricular cannulation

Intracerebroventricular (ICV) cannulations were performed under anesthesia and aseptic conditions. The right lateral cerebral ventricle was accessed. The rats, weighing between 250 and 350 g, were anesthetized intra-muscularly with 0.2 ml/100 g of a mixture composed of acepromazine (5 mg (0.5 ml) of 10 mg/ml), ketamine (150 mg (1.5 ml) of 100 mg/ml and xylazine (30 mg (1.5 ml) of 20 mg/ml). The dorsum of the head was shaved and rats were placed in a stereotaxic holding device. The skin was disinfected with 70% isopropyl alcohol and a 22-gauge guide cannula (c313G – Single guide cannula, Plastics One, Inc., Roanoke, VA), cut to a length of 15 mm, was implanted into the right lateral ventricle. The stereotaxic coordinates used were with respect to the bregma, AP = -0.8 mm, *L *= -1.4 mm and depth = 15 mm below the skull surface. The cannula was held in place with three stainless-steel screws and dental cement on the skull. A 32-gauge dummy cannula (c313DC – Single guide cannula, Plastics One, Inc.) cut at 12 mm was inserted when the rat was not receiving an injection. Rats were allowed 1 week to recover, when they were expected to recover their initial body, presented when the day the surgery was performed. The rats were divided into 3 groups and sterile saline solution, LS solution, and 0.8 M lactic acid solution were administered.

### ICV injection

One week after the establishment of ICV cannulation, 0.2 μl of saline solution (n = 9), 0.8 M lactic acid solution (n = 7) or LS (n = 22) were injected using a 10 μl Hamilton syringe connected to a catheter with a 28-gauge internal cannula (c313I – Internal cannula, Plastics One, Inc.) cut at 15 mm. The rats were kept for up to 120 hr and were monitored for changes in behavior, body weight, temperature, and food and water consumption at 0, 12, 24, 48, 96 and 120 hr after ICV injection. Rats receiving each solution were euthanized by CO_2 _inhalation after 12, 24, 48, and in selected cases 120 hr after the ICV injection. The entire brain, intestines and retroperitoneal adipose tissue were collected immediately after euthanasia, fixed in 10% buffered formalin for 24 hr and processed routinely for histopathology.

Thirty eight young Sprague-Dawley male rats were divided into 3 groups to study the potential effect of injection of LS into the cerebral ventricles on angiogenesis of CNS blood vessels. The experimental group (22) was administered LS. One control group (9 rats) received saline solution. The second group (7 rats) received lactic acid to exclude the possibility that low pH (the pH of LS is 2.35) rather than specific components of LS was responsible for any ensuing changes. The osmolality of the LS (320 mOsm) and lactic acid (250 mOsm) solution was comparable. The osmolality had no effect in our *in vitro *experiments [5,6] as it was immediately diluted at 200 ×. In the *in vivo *experiments the dilution factor was likely comparable, and we never noticed any immediate effect, including focal necrosis, on morbidity of the animals in this study or in our previous report describing angiogenesis *in vivo *[5]. In the saline solution control group three rats were injected and euthanized at 12 hr, and two rats at 24, 48, and 120 hr in each experiment. In the lactic acid control group 2 rats were injected and euthanized at 12, 24, and 48 hr, and one rat was euthanized at 120 hr post injection. In the LS group, 5 rats were euthanized at 12 hr, 4 rats at 24 hr, 4 rats at 48 hr, 1 rat at 96 hr, and 8 rats at 120 hr. Body weight and other indices were determined in all rats at intervals of 12, 24, 48, 96 and 120 hr after ICV injections until their euthanasia.

### Immunohistochemistry

For immunohistochemistry a polyclonal antibody raised in rabbits against the N-terminal region of leptin, the product of the *ob *gene (Ob H-146, Santa Cruz Biotechnology, Santa Cruz, CA, USA) was used as primary antibody at 1:500 dilutions for 1 hr at 37°C after antigen retrieval was performed in microwave for 10 min in Antigen Unmasking Solution (Vector Laboratories, Burlingame, CA, USA). The secondary antibody (anti-rabbit made in goat from Roche Applied Sciences, Indianapolis, IN, USA) was used in a 1:1000 dilution for 1 hr at 37°C. The slides were then incubated with avidin-biotin complex (ABC) method using Vectastain ABC kit (Vector) for 1 hr at 37°C.

Monoclonal rat antibody towards tumor necrosis factor α (TNFα) (ab11564 from Abcam, Cambridge, MA, USA) was used at 1:500 dilution, and monoclonal rat antibody towards granulocyte-macrophage colony stimulating factor (GM-CSF) (MAB5181, R&D Systems, Minneapolis, MN, USA) was used at 1:100 dilution. Anti-rat IgG from Vector was used as a secondary antibody for both antibodies at 1:250 for 1 hr at 37°C followed by color detection using ABC-AP kit and alkaline phosphatase substrate SK-5100 kit, both from Vector.

### In situ hybridization assay

The riboprobe used for in situ hybridization was based on the sequence of the rat leptin gene (Genbank accession number D49653). A pair of primers was designed to create a 422 bp long probe:

sense: 5'-TGT TCA AGC TGT GCC TAT CCA CAA AGT CCA GGA-3'

antisense: 5'-GAA GAA TGT CCT GCA GAG AGC CCT GCA GCC TG-3'

The mRNA was extracted from fresh rat fat with Trizol following manufacturer's instructions (Quiagen, Valencia, CA, USA). The first strand of cDNA was synthesized using Superscript III kit (Invitrogen, Carslbad, CA, USA) followed by RT-PCR. After purification from an agarose gel the PCR products were ligated into the TA- vector (pGEM-Teasy, Promega, Madison, WI) and ligation products were introduced into E. coli by heat-shock. Positive colonies were confirmed by DNA sequencing performed by The Sequencing and Synthesis Facility at University of Georgia. A confirmed positive colony (it is in a reversed direction in pGEM-Teasy) was cultured and plasmid DNA prepared using a Promega mini-prep kit. The resulting constructs were cleaved with restriction enzyme Sac*I*. This was followed by *in vitro *transcription with T7 RNA polymerase to generate an anti-sense RNA of approximately 422 bp nucleotides in length. Those anti-sense riboprobes were used for *in situ *hybridization of formalin-fixed, paraffin-embedded tissue samples to detect the production of leptin in rat tissues.

Forward primer: 5'-TGT TCA AGC TGT GCC TAT CCA CAA AGT CCA GGA-3' (sense)

Reverse primer: 5'-GAAGAATGTCCTGCAGAGAGCCCTGCAGCCTGC-3' (antisense)

For *in situ *hybridization the slides were first heated at 70°C for 10 min and deparafinized in Hemo-De. Tissue sections were allowed to dry and then were rehydrated in PBS + 5 mM MgCl_2_. Slides were incubated in Proteinase K (100 μl/ml) diluted in proteinase K buffer (10 mM Tris pH 7.5 + 2 mM CaCl_2_). The enzymatic reaction was stopped with 0.1 M glycine + 0.2 M Tris, pH 7.5. Prehybridization solution was added to sections for 1 hr (42°C). This was followed by the addition of the probe (3 μl/slide) mixed with the prehybridization solution. A total of 70 μl of probe + prehybridization solution/slide was used and applying direct onto a siliconized coverslip, which was sealed with nail hardener and kept in humid chamber overnight (42°C).

The next day, after several washes the slides were incubated with anti-dig AP diluted 1:300 in 2% normal serum sheep in buffer1(150 mM NaCl + 100 mM Tris, pH 7.5) for 2 hr. Slides were washed in buffers 1 and 3 (100 mM NaCl + 100 mM Tris+ 50 mM MgCl_2_, pH 9.5) for 5 min. Substrate (NBT + BCIP) was added and slides were checked for staining. On average, the indicator color was detected in brain tissues after 40 min and in adipose tissue after 3 hr.

### Western blotting

Intestines and retroperitoneal adipose tissues collected from all three experimental groups were homogenized in buffer composed of 20 mM Tris, 5 mM EDTA, 2 mM Na_3_NO_4_, 10% glycerol, 1% Triton X-100 and 1 mM PMSF. Aliquots of 10 μg protein were loaded per lane and separated in 8% SDS-polyacrylamide gels, then transferred on nitrocellulose membrane and with a polyclonal antibody raised in rabbits against the N-terminal region of leptin, the product of the *ob *gene (Ob H-146) or a polyclonal antibody raised in rabbits against the leptin receptor (Ob-R H-300, both from Santa Cruz Biotechnology) at 1:1000 dilutions. Antibody – antigen complexes were detected by DAB kit (Vector).

### Statistical analysis

One-way analysis of variance test using the General Linear Models procedure of SAS was employed to test overall differences between initial weight and weight at euthanasia within LS-treated groups.

## Results

**Figure 1:**
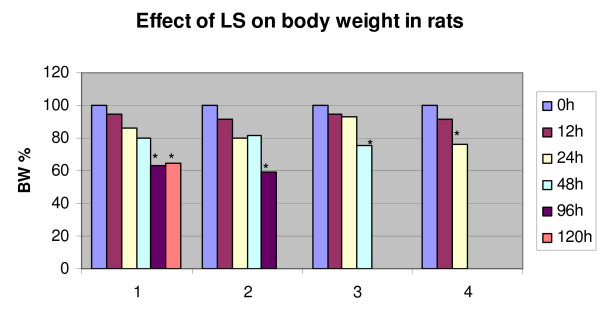
**Average body weight of rats after intracerebroventricular injection**. ICV injection of LS led to statistically significant decrease in body weight 48 hr and later after a single ICV injection. The groups 1–4 represent four experiments. *time points significantly different from 0 h at α = 0.05.

Because our previous data have shown that topical administration of LS led to influx of inflammatory cells into skin wound sites and to stimulation of angiogenesis in rodent skin and subcutaneous tissues [5], we tested LS for the ability to induce angiogenesis in the rat brain. No angiogenesis was observed in brains of any of the rats, be it the treatment or control groups. Neither was influx of inflammatory cells noted previously in subcutaneous tissues [5] present anywhere in CNS. Instead the injection of LS elicited reductions in body-weight. In comparison with the control groups, the animals that received *Lactobacillus *supernatants had a 24% decrease in body weight within 48 hours, and were able to sustain it at least up to 120 hr (Table 1). A slight decrease in the body weight was observed in the animals that received lactic acid (4.4%). Because we did not have enough control rats for a statistical analysis at later time points (at 96 and 120 hr) we did not perform it for evaluation body weight loss among the three groups. However, Table 1 reveals a clear trend of loss of body weight in LS-treated rats, but not in the two control groups. One-way analysis of variance was used to evaluate body weight loss within the LS-treated group (Fig. 1). In this analysis body weight was measured on all surviving rats at time points before their euthanasia. Our data revealed significant difference in body weight between 0 and 48 hr in rats treated with LS at α = 0.05 level (P-value = 0.084), between 0 and 96 hr (P-value = 0.0025) and between 0 and 120 hr (P-value = 0.0064).

**Table 1:** Comparison of weight between control and LS-treated rats.

Time	0 h	12 h	24 h	48 h	96 h	120 h
Rats ID BW%						
C1-C9 (n = 9)	100	99				
C1-C6 (n = 6)	100	98.3	98.3			
C1-C4 (n = 4)	100	98.3	98.7	95.6		
C1-C2 (n = 2)	100	97.4	97.4	98.6	95.9	97.3
LA1-LA7 (n = 7)	100	97.7				
LA1-LA5 (n = 5)	100	98.5	99.4			
LA1-LA3 (n = 3)	100	98.5	98.3	99.5		
LA1 (n = 1)	100	95.7	95.7	95.9	100.8	10.3.1
LS1-LS22 (n = 22)	100	94.9				
LS1-LS17 (n = 17)	100	91.8	75.8			
LS1-LS13 (n = 13)	100	94.8	92.9	75.1		
LS1-LS8 (n = 9)	100	91.3	80.2	81.4	59.4	
LS1-LS8 (n = 8)	100	94.9	85.8	79.8	63.1	65

• Control rats labeled C1-C9 received 0.2 μl of saline solution, rats C7-C9 were euthanized at 12 hr; C5-C6 were euthanized at 24 hr; C3-C4 were euthanized at 48 hr; C1-C2 were euthanized at 120 hr.

• Rats labeled LA1-LA7 received 0.2 μl of 0.8 M lactic acid solution; LA6- LA7 were euthanized at 12 hr; LA 4- LA 5 were euthanized at 24 hr; LA 2- LA 3 were euthanized at 48 hr; LA 1 was euthanized at 120 hr.

• Rats labeled LS1-LS7 received 10 μl of LS, rats LS18- LS22 were euthanized at 12 hr; LS14- LS17 were euthanized at 24 hr; LS10- LS13 were euthanized at 48 hr; LS9 were euthanized at 96 hr; LS1-LS8 were euthanized at 120 hr.

**Figure 2:**
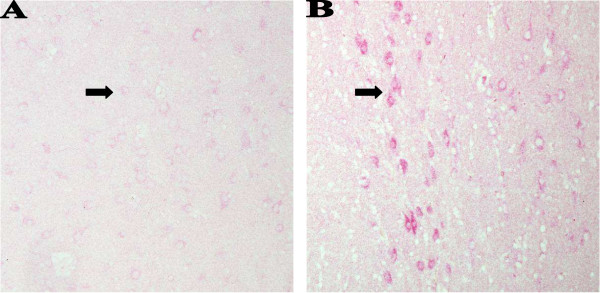
**Immunohistochemistry for leptin in brain tissue**. Rats injected with saline solution, lactic acid or LS were euthanized 48 hr after injections and their brains were fixed in formalin immediately after euthanasia. Cytoplasmic immunostaining for leptin (arrows) was stronger in neurons of thalamus in LS-injected animals (B) than in corresponding cells of rats injected with saline (A). No counterstain. Magnification × 200.

**Figure 3:**
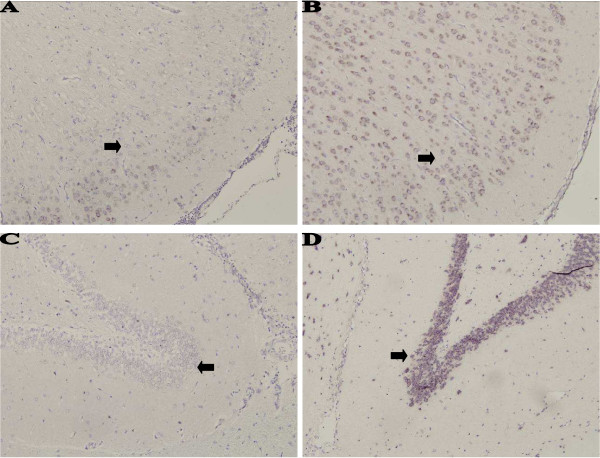
***In situ *hybridization for leptin**. Rats injected with saline solution, lactic acid or LS were euthanized 48 hr after injections and their brains were fixed in formalin immediately after euthanasia. Leptin mRNA was visualized using *in situ *hybridization in sections of the brains of rats injected with saline solution (A, C), and LS extract (C, D). Leptin expression is observed in the cytoplasm of neurons in the hypothalamus (A, B; arrows) and hippocampus (C, D; arrows). Stronger expression is noted in LS-injected animals. Light counterstain with hematoxylin. Magnification × 200.

**Figure 4:**
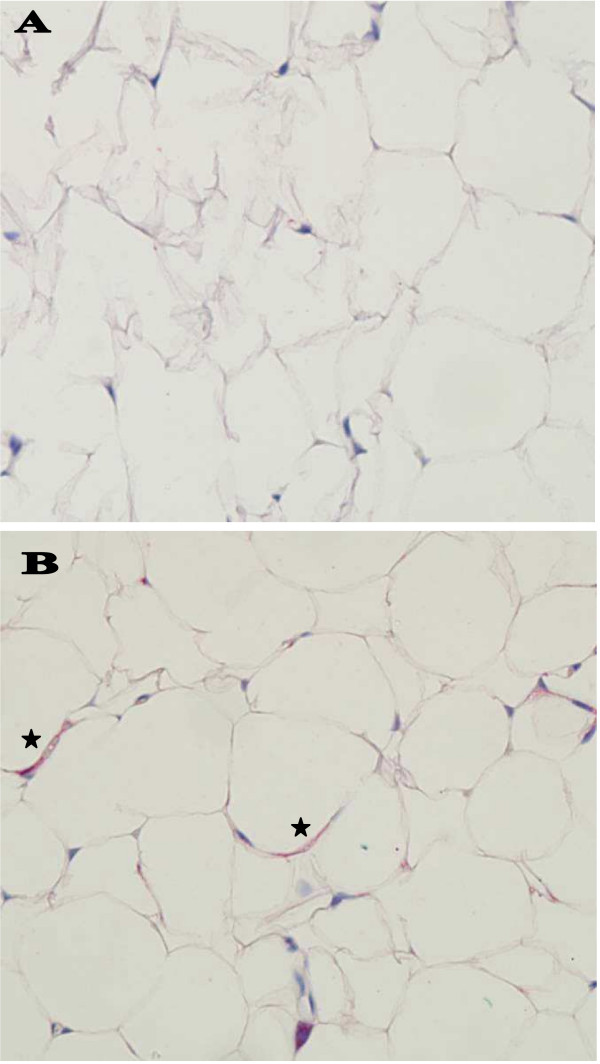
**Immunohistochemistry for leptin in adipose tissue**. Rats injected with saline solution, lactic acid or LS were euthanized 48 hr after injections and the retroperitoneal adipose tissue was fixed in formalin immediately after euthanasia. Cytoplasmic immunostaining for leptin (*) was stronger in adipocytes in LS-injected animals (B) than in corresponding adipocytes of rats injected with saline (A). Light counterstain with hematoxylin. Magnification × 400.

Because LS caused marked increase in leptin production in bovine endothelial cells and because leptin is involved in regulation of body weight we hypothesized that LS might also regulate leptin expression in the brain. We employed both immunohistochemistry and *in situ *hybridization to see whether LS injection leads to leptin overexpression in the brain. Leptin expression was studied in multiple brain sections of control and LS-injected rats. Strong cytoplasmic stain was observed by both techniques in neurons of the cerebral cortex, thalamus (Fig. 2), hypothalamus and hippocampus (Fig. 3), and weaker staining in the cells of the choroid plexus (data not shown). Control animals demonstrated much less intense staining in neurons (and other cells) located in the same regions and tissues using immunohistochemistry and almost no staining with *in situ *hybridization technique. Peripheral adipocytes exhibited light immunostaining for leptin in LS-injected rats (Fig. 4). Immunoblotting of protein extracts from intestines and from retroperitoneal adipose tissue from LS-treated and control rats showed only mild increase in both leptin and leptin receptor (data not shown).

To see whether the effect on leptin synthesis was specific we also performed immunohistochemistry for GM-CSF and TNFα in CNS sections. In contrast no immunohistochemical staining for GM-CSF and TNFα was observed in brains from control and treated rats (data not shown), though LS stimulated production of TNFα by several cell lines [5]. Such results indicate that the effect of LS on leptin expression is specific.

## Discussion

We have shown that injection of LS into rat cerebral ventricles led to decrease in body weight. This reduction was independent of food and water intake and it occurred concomitantly with a strong increase in leptin expression in neurons of the cortical region, thalamus, hypothalamus, and hippocampus. Mild increase in leptin was noted in the cells of the choroid plexus of the rat brain, and peripheral tissues such as intestines and fat. This finding was in contrast with our previous studies where local administration of LS led to angiogenesis and influx of inflammatory cells [5]. Interestingly, in a separate series of experiments LS stimulated leptin secretion into medium conditioned by bovine endothelial cells exposed to *Lactobacillus *supernatants. We hypothesize that the kind of biological activity exerted by leptin depends on the tissue or cell type serving as an LS target. It is also possible that differential expression of other cytokines, induced by LS in CNS and not yet identified, play a crucial role in modulating the effect of LS on body weight.

Several studies showed variable effects of *Lactobacillus *extracts on the production of leptin or its plasma level. The main reason for the discrepancy is the route of administration (ours is the only study where a *Lactobacillus *preparation was administered directly into CNS), *Lactobacillus *strain and/or animal species or breed used. Bleau et al. [1] described *Lactobacillus*-induced decrease in leptin release by adipocyte derived from SJL mice, whereas the same *Lactobacillus *preparation led to an increase in leptin release when used to treat C57BL/6 adipocytes. They ascribed this to regulation of leptin synthesis by IL1β and TNFα produced by C57BL/6 macrophages upon stimulation by *Lactobacillus *preparation. In another report probiotic capsules containing *L. acidophilus *and *Bifidobacterium longum *had no effect on plasma leptin in a group of men even after 2 months of oral intake [14]. Oral administration of *L. plantarum *to smokers led to decrease in plasma concentrations of leptin and other parameters which was attributed to anti-inflammatory properties of *L. plantarum *[15]. In contrast, our preparations of *L. acidophilus *do promote a variety of pro-inflammatory processes [5,6]. Some of these processes (e.g., angiogenesis), though not all, might be secondary to increased leptin expression. Our previous data have shown that LS stimulates TNFα production [5] which in turn would lead to increased leptin expression [1]. This mechanism would be less likely to be operational in CNS as immunohistochemistry did not identify any TNFα in CNS of rats after LS injection in our experiments.

We chose to look at the presence of GM-CSF and TNFα (neither cytokine was included in the angiogenesis antibody array used by us) in the rat brain for several reasons. GM-CSF was described to suppress food intake and reduce body weight in rodents after intraventricular injection [16]. In our experiments we found that food intake of LS-injected rats was comparable to food intake of control animals and that no GM-CSF was present in rat CNS using immunohistochemistry. TNFα, also known as cachectin, leads to sometimes a significant weight loss. As mentioned above LS stimulates the production of TNFα in peripheral macrophages [5] but not in CNS.

That leptin is primarily synthesized by adipocytes has been known for many years [17]. Less well known but well documented by many investigators has been the production of leptin in the brain. For example, Wiesner et al. have shown that leptin is released from the brain, particularly in obese men [18]. Similarly to our findings, immunostaining has localized leptin to many areas of the brain: cortex, cerebellum, hypothalamus and hippocampus [19]. In hypothalamus, leptin regulates energy intake and expenditure [20], though some experiments point to leptin signaling in caudal brainstem neurons as transmitting gastrointestinal signals indicating satiety [21]. However, leptin during fetal and neonatal period also modulates brain development, especially in the hippocampus [22,23] and hypothalamus [17]. It is thought that leptin stimulates the proliferation and differentiation of neural cell during early life as brain weight and DNA and protein content are reduced in *ob/ob *(leptin-deficient) mice [24].

## Conclusion

In conclusion, this study shows that direct administration of LS into the brains of rats resulted in weight loss without a decrease in food consumption. This was accompanied an increase in leptin expression in neurons and peripheral adipose tissues. The advantage (and a possible disadvantage because of potential side effects) of LS use over purified leptin is the possibility that active components of LS modulate the expression or release of other peptides involved in metabolism and body weight control. The identification and large scale testing of these compounds is currently under investigation in our laboratory.

## Competing interests

JH, SJL and WOL are holding a provisional patent application describing the effects of LS. They have not received any financial reimbursement or fees. They have no other competing interests.

## Authors' contributions

RS participated in experiment design, performed the animal experiments, immunohistochemistry and *in situ *hybridization and wrote the manuscript. JH, SJL and WOL conceived and designed the study, and supervised the experiments. JZ performed the cell culture, angiogenesis assay, immunoblotting, statistical analysis and participated in immunohistochemistry and *in situ *hybridization. All authors read and approved the final manuscript.

## Pre-publication history

The pre-publication history for this paper can be accessed here:


